# Randomized clinical trial: the effect of probiotic *Bacillus coagulans* Unique IS2 vs. placebo on the symptoms management of irritable bowel syndrome in adults

**DOI:** 10.1038/s41598-019-48554-x

**Published:** 2019-08-21

**Authors:** Ratna Sudha Madempudi, Jayesh J. Ahire, Jayanthi Neelamraju, Anirudh Tripathi, Satyavrat Nanal

**Affiliations:** 1 Centre for Research & Development, Unique Biotech Ltd., Plot No. 2, Phase-II, Alexandria Knowledge Park, Hyderabad, Telangana 500078 India; 2 Life Veda Treatment and Research Centre, Worli, Mumbai 400030 India; 3 Nanal Clinic, Anand Bhuvan, Gore wadi, Mahim (W), Mumbai, 400016 India

**Keywords:** Irritable bowel syndrome, Microbiota

## Abstract

The therapeutic effects of *B*. *coagulans* Unique IS2 have been well established in children with irritable bowel syndrome (IBS), but its efficacy in adults remain under reported. Thus, in this study the efficacy of *B*. *coagulans* Unique IS2 in the management of IBS symptoms in adults was investigated. Patients (*n* = 153) fulfilling Rome III criteria were provided placebo capsules for a 2 weeks run-in period. Only patients satisfying compliance criteria (*n* = *136*) were randomized (double blind) to receive either *B*. *coagulans* Unique IS2 (2 billion CFU) or placebo capsules daily for 8 weeks. Reduction of abdominal discomfort/pain intensity and increase in complete spontaneous bowel movements were analyzed as primary end points. Other clinical symptoms of IBS and serum cytokines were also evaluated. *B*. *coagulans* Unique IS2 showed significant improvement in primary and secondary endpoints, as compared to placebo. Haematology of both the arms remained normal. No significant changes in pro- (IL-6, IL-12, TNF-α, INF- γ) and anti-inflammatory cytokine (IL-10) levels were detected at the end of *B*. *coagulans* treatment (8 weeks) as compared to placebo. *B*. *coagulans* was well tolerated with no severe adverse events to report. Overall, the results demonstrate that *B*. *coagulans* Unique IS2 is efficacious in the management of IBS symptoms in adults (18–60 years).

## Introduction

Irritable bowel syndrome (IBS) is a chronic gastrointestinal tract disorder known to cause severe abdominal pain due to changes in normal gut behavior^1,2^. According to Rome IV diagnostic criteria, IBS is defined as a functional bowel disorder (FBD) in which patient reports recurrent abdominal pain on an average at least one day in a week in the last 3 months, associated with two or more symptoms such as pain during defecation, change in stool frequency and stool form^2,3^. Based on stool patterns, IBS is classified into four subtypes; (i) constipation predominant (IBS-C), (ii) diarrhea predominant (IBS-D), (iii) mixed bowel habits (IBS-M), and (iv) un-subtyped (IBS-U)^3^. The estimated global prevalence of IBS is very high at 11% affecting individuals of all ages^4^. Despite the high number of cases and economic burden on society, IBS is one of the ignored FBDs^4,5^.

The pathogenesis of IBS is one of complex, multifactorial processes in which diet, bile, enteric infections, antibiotic treatment, gender and psychosocial condition play an important role in altering normal gastrointestinal functions^6–9^. The diagnosis of IBS is difficult due to change in symptoms over time and close resemblance of symptoms to other disorders (lactose or fructose intolerance), and absence of specific biomarkers for IBS detection^2^. Besides this, the therapeutic options available for IBS are limited and ineffective due to complex and diverse ways of disease development^10^.

Probiotics are live microorganisms, which when given in sufficient amounts confer a health benefit on the host^11^. Recently, the use of probiotics in the management of dysbiosis and or stabilization of the host microbiota in IBS is gaining lot of interest^12^. The proposed mechanism of probiotics action in IBS is undefined but may include inhibition of colonization of pathogens, support of intestinal barrier integrity and function, production of beneficial micronutrients, and activation and augmentation of the enteric nervous system^1^. The benefits of probiotics however rely on strain, delivery of sufficient amount of active cells and duration of therapy^13^.

Nowadays, spore forming bacteria are widely used in commercial probiotic formulations due to their outstanding probiotic properties, survival through various industrial processes, and room temperature stability over non-sporulating probiotics^14–16^. Like well-known non-sporulating probiotic cultures, spore producing probiotics are reported to produce a range of proteins, peptides, enzymes, antimicrobial substances, vitamins, exo-polysaccharides and carotenoids, and possess biotherapeutic potential for the betterment of host^16^. The spore producers are known for their efficacy in treatment of *Helicobacter pylori* infection, gingivitis, constipation, diarrhoea and maintaining intestinal homeostasis^7,12,16^. However, the role of these probiotics in the management of IBS has rarely been investigated^17–21^. In our previous study, we have shown that *B*. *coagulans* Unique IS2 supplementation reduced abdominal pain, discomfort and disease severity and improved the stool consistency in children (4–12 years) with IBS^22^. In the present double blind, randomised controlled study, we investigated the effect of *B*. *coagulans* Unique IS2 supplementation on abdominal pain, complete spontaneous bowel movement (CSBM) and disease severity in adults (18–60 years) with irritable bowel syndrome (IBS).

## Results

### Screening and other evaluations

**Figure 1:**
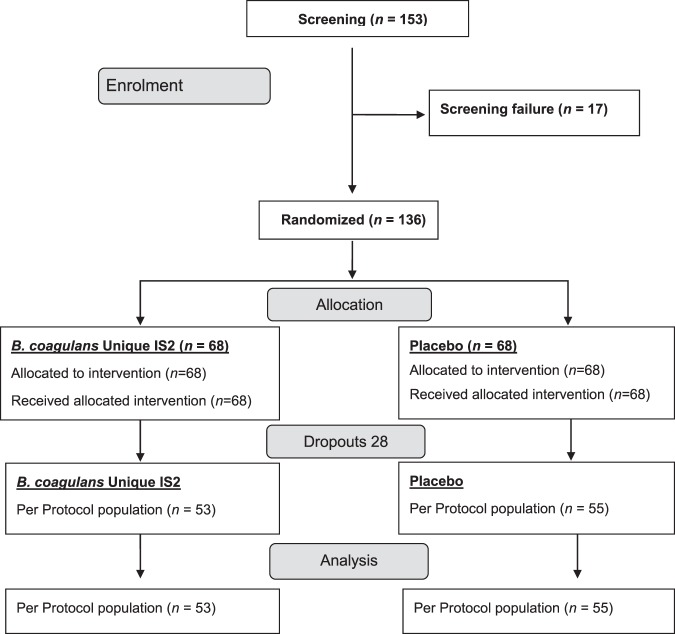
Study flow chart.

One fifty-three patients were screened for IBS, out of which 136 patients showed 80% compliance to the presence/persistent trial entry criterion (2 week screening). The patients were randomized into two groups- the probiotic and placebo treated groups in the ratio 1:1. Out of 136 patients, only 108 (*n* = 53: *B*. *coagulans*; *n* = 55: placebo) completed the study (per protocol (PP) population). The remaining 28 patients (intention to treat (ITT) population) dropped out due to protocol deviations (11), violations (2) and unavailability during follow-up visits (15) (Fig. 1). The first patient was enrolled in July 2016 and last completed the study in August 2017. Both the arms of study i.e. *B*. *coagulans* Unique IS2 and placebo showed comparable baseline demographic characteristics (Table 1). The patients mean age in *B*. *coagulans* Unique IS2 group was 44.4 years and 42.3 years in the placebo group. 27.28% patients were females and 72.22% were males. Mean complete spontaneous bowel movements (CSBMs) score observed at baseline was 2.5 (*B*. *coagulans*) and 2.2 (placebo) (*p* = 0.2825). The mean baseline abdominal discomfort score was 3.6 for both the groups (*p* = 0.5306) (Table S1). All subjects in the study were of Indian origin.

**Table 1:** Patients baseline demographic characteristics.

	*B*. *coagulans* (*n* = *53*)	Placebo(*n* = *55*)	Total (*n* = 108)	*p* value
**Gender** ***n*** **(%)**				
Male	41 (77.36)	37 (67.27)	78 (72.22)	
Female	12 (22.64)	18 (32.73)	30 (27.78)	0.2421*
**Age (years)**				
Mean	44.4	42.3	43.4	
Median	46.0	42.0	45.0	
(min/max)	(20/60)	(21/60)	(20/60)	0.3734
**Height (cm)**				
Mean	160.6	157.9	159.2	
Median	161.0	158.0	160.0	
(min/max)	(110/180)	(137/185)	(110/185)	0.1016
**Weight (kg)**				
Mean	63.1	62.8	62.9	
Median	60.0	61.0	61.0	
(min/max)	(42/105)	(38/86)	(38/105)	0.8900

*p* value: inter group (chi-square* and two sample *t* test).

95.37% patients had a history of abdominal pain, bloating, infrequent stools, passage of gas, straining and incomplete evacuation. 15.75% patients reported acidity, hyperacidity, constipation, musculoskeletal, connective tissue and hepatobiliary system disorders (Table S2). Apart from this, *B*. *coagulans* and placebo group showed comparable values in terms of allowed prior medication usage (Table S3). More than 80% drug compliance was observed in both the treatment groups. None of the patients were withdrawn due to non-compliance of investigation product.

### Primary and secondary efficacy evaluations

**Figure 2:**
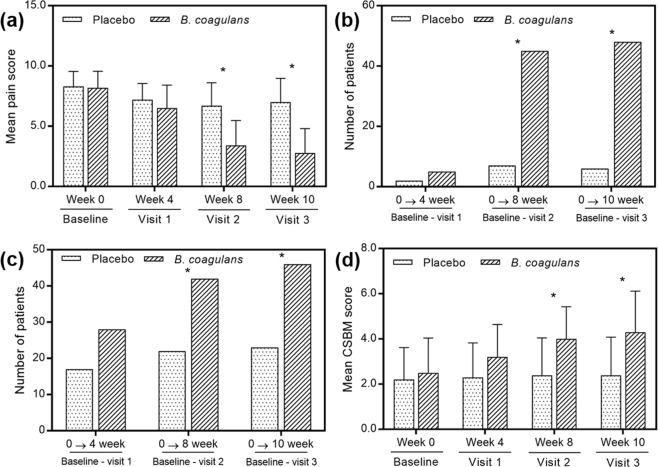
Effect of *B*. *coagulans* and placebo on (**a**) abdominal pain intensity reported on 0–10 scale, (**b**) number of patients reporting more than or equal to 50% pain reduction, (**c**) number of patients reporting more than or equal to 1 CSBM and, (**d**) mean CSBM score. p* < 0.0001.

The pain intensity scores were reduced in *B*. *coagulans* Unique IS2 treated group as compared to placebo and baseline (Fig. 2a). At the end of week 8, the mean score of baseline pain was reduced from 8.2 ± 1.37 to 3.4 ± 2.08 in *B*. *coagulans* Unique IS2 treated group; whereas, in placebo it decreased from 8.3 ± 1.25 to 6.7 ± 1.92 (two sample *t*-test, *p* < 0.001). Patients showing ≥50% pain reduction from their baseline visit were considered as responders in the study. The comparative analysis showed that at 4^th^ week, 5 (9.43%) patients of *B*. *coagulans* Unique IS2 group (*n* = *53*) and 2 (3.64%) patients of placebo (*n* = *55*) had ≥50% pain reduction (chi-square test, *p* < 0.2212). Later at 8^th^ week, 45 (84.91%) patients of *B*. coagulans Unique IS2 group (*n* = *53*) and 7 (12.73%) patients of placebo (*n* = *55*) demonstrated ≥50% pain reduction (chi-square test, *p* < 0.001). A similar trend was observed at the 10^th^ week, 48 patients (90.57%) of *B*. *coagulans* Unique IS2 group (*n* = *53*) and 6 patients (10.91%) of placebo (*n* = *55*) showed ≥50% pain reduction (chi-square test, *p* < 0.001) (Fig. 2b).

After 8 weeks of treatment, significantly higher (two sample *t*-test, *p* < 0.001) responder rate of CSBM was observed in 42 patients (79.25%) of *B*. *coagulans* Unique IS2 group (*n* = *53*) as compared to 22 in placebo (40.00%; *n* = *55*) (Fig. 2c). The mean baseline CSBM score of *B*. *coagulans* Unique IS2 group was increased from 2.5 ± 1.54 to 4.0 ± 1.43 and in placebo group from 2.2 ± 1.42 to 2.4 ± 1.65 (Fig. 2d).

**Figure 3:**
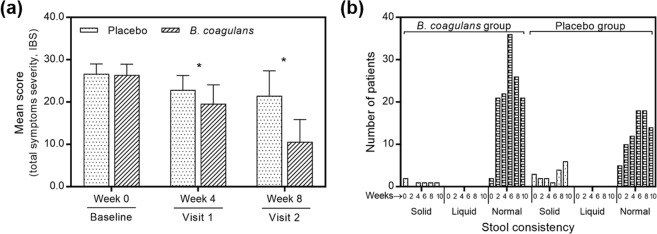
Effect of *B*. *coagulans* and placebo on (**a**) mean score of total symptoms severity of IBS and, (**b**) stool consistency. p* < 0.0001.

Patients of *B*. *coagulans* Unique IS2 group showed significant reduction in all (eight) domains of severity symptoms as compared to placebo during 4^th^ and 8^th^ week follow-up. At 8^th^ week, the mean baseline abdominal discomfort domain score was reduced (*p* < 0.0001) from 3.6 ± 0.60 to 1.3 ± 0.85 in *B*. *coagulans* Unique IS2 group and from 3.6 ± 0.56 to 2.8 ± 0.87 in placebo group. Similarly, the mean baseline total severity symptoms score of *B*. *coagulans* group decreased (*p* < 0.0001) from 26.4 ± 2.54 to 10.6 ± 5.26 and 26.7 ± 2.31 to 21.5 ± 5.88 in placebo group (Fig. 3a). From 5^th^ week onwards the patients of *B*. *coagulans* group showed significant (*p* < 0.0001) improvement in abdominal discomfort, bloating, urgency, incomplete evacuation, straining, passage of gas, bowel habit satisfaction, overall assessment of IBS symptoms and total score (Table S4). The stool consistency of *B*. *coagulans* Unique IS2 group significantly (*p* < 0.001) improved after 6 weeks of treatment, with 65% (36 out of 53) of patients gaining normal stool consistency. However, only 32.72% (18 out of 55) of patients achieved normal stool consistency in placebo (Fig. 3b).

**Figure 4:**
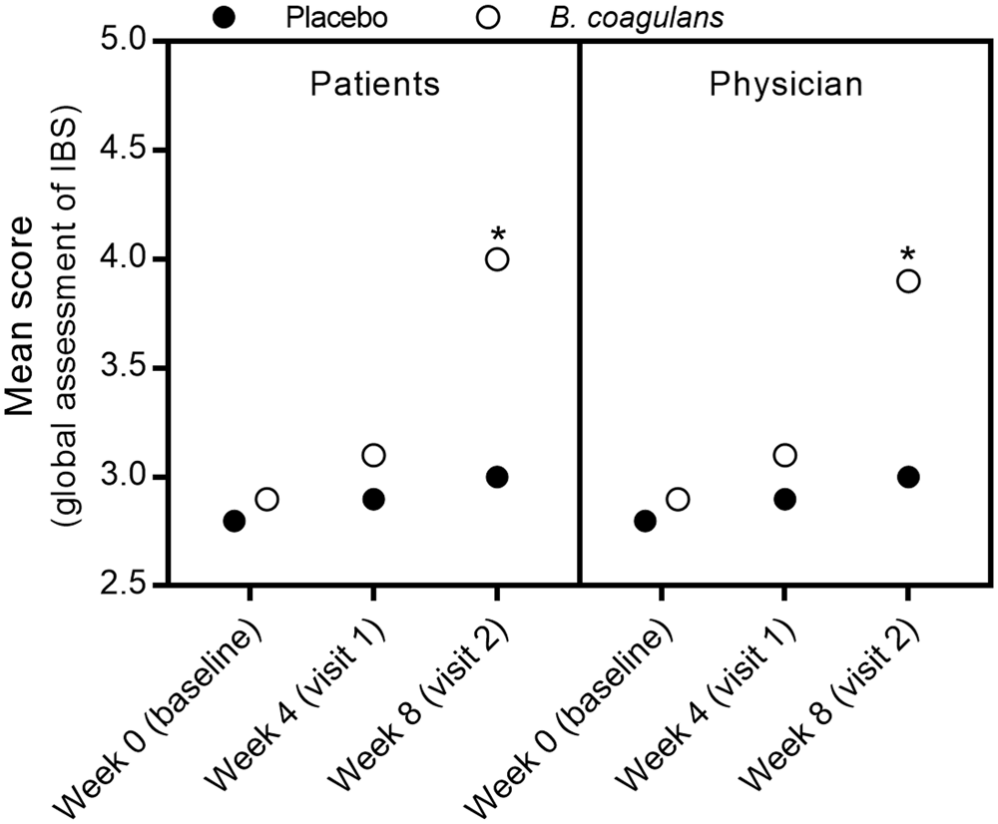
Effect of *B*. *coagulans* and placebo on patients and physician global assessment of IBS. Results are reported on 1–5 scale. p* < 0.0001.

The results of patient global assessment of *B*. *coagulans* group (*n* = *53*) indicated that 10 (18.87%) patients had complete and 34 (64.15%) had considerable relief from IBS symptoms (Fig. 4). The percentage was significantly (*p* < 0.0001) higher as compared to placebo (*n* = *55*; 0 (0%) complete and 6 (10.91%) considerable relief). Furthermore, physician global assessment revealed that 11 (20.75%) patients in *B*. *coagulans* group (*n* = *53*) had complete and 31 (58.49%) had considerable relief from symptoms of IBS as compared to 0 (0%) and 6 (10.91%) in placebo (*n* = *55*) (Fig. 3). The difference recorded was statistically significant (*p* < 0.001). No significant changes were observed in serum TNF-α, IFN-γ, IL-6, IL-10 and IL-12 levels with *B*. *coagulans* Unique IS2 treatment as compared with placebo (Table 2).

**Table 2:** Serum cytokine profile (TNF-α, IFN-γ, IL-6, IL-10 and IL-12) of participants.

Visit	*B*. *coagulans*				Placebo				Absolute change from baseline				*p* value**	
	*n*	mean	SD	(min/max)	*n*	mean	SD	(min/max)	mean	SD	CI	*p* value*	*B*. *coagulans*	Placebo
TNF-α: baseline	53	23.8	94.9	(0.29/762)	55	15.3	46.3	(0.6/377)						
TNF-α: week 8	53	28.8	94.6	(1.13/695)	55	16.8	20.9	(4/141)	1.5	38.11	−5.77–8.77	0.7613	0.7785	0.8148
IL-6: baseline	53	5.0	15.4	(0.087/123.9)	55	4.2	7.2	(0.1/53.6)						
IL-6:week 8	53	2.6	1.9	(0/8.69)	55	4.8	11.03	(0.1/81.8)	−1.1	15.53	−4.05–1.87	0.2555	0.2375	0.7117
IL-10:baseline	53	19.3	10.5	(0.33/48.8)	55	14.9	9.4	(0.44/46.3)						
IL-10: week 8	53	21.8	13.9	(4.33/96.3)	55	21.6	9.7	(2.24/44.3)	5.1	11.54	2.90–7.30	0.1015	0.2821	0.0002
IL-12: baseline	53	288.5	107.7	(128.33/678.3)	55	320.6	123.4	(186.67/661.6)						
IL-12: week 8	53	311.5	108.8	(120/618.2)	55	333.3	134.7	(136.04/681.1)	11.5	150.06	−17.09–40.16	0.6798	0.2562	0.5928
INF- γ: baseline	53	6.7	18.05	(0.42/147)	55	5.3	4.2	(0.29/30.2)						
INF- γ: week 8	53	5.7	5.01	(0.03/39.5)	55	6.4	10.2	(0.94/80.1)	1.0	8.78	−0.64–2.71	0.9996	0.6695	0.4528

*p* value*: inter group (two sample *t* test). *p* value**: intra group (two sample *t* test). *n*: number of patients. Values are represented in pg/mL (picograms per millilitre).

The usage of rescue medication in *B*. *coagulans* Unique IS2 group was lower than placebo. Only 2 (3.77%) patients of *B*. *coagulans* group (*n* = *53*) used rescue medication as compared to 7 (12.72) in placebo (*n* = *55*). Totally, 6.48% patients used Duphalac syrup, 0.92% Loperamide and Lomofen tablets.

### Safety evaluations

No adverse events were recorded in *B*. *coagulans* Unique IS2 group and placebo. The results of heamatological analysis, physical and vital examinations revealed no abnormal findings between both the arms and their baseline values (Tables S5, 6 and 7).

## Discussion

*Bacillus coagulans* Unique IS2^TM^ (MTCC 5260, ATCC PTA-11748) is a spore forming, non-toxic commercial probiotic strain^23,24^. The clinical trials conducted on anti-hypercholesterolemic effect^25^, liver cirrhosis^26^, bacterial vaginosis^27^, acute-diarrhea^28^, abdominal pain^29^, constipation^30^, oral health^31^ and IBS in children^22^ have proven therapeutic efficacy and safety of this strain. Moreover, *in vitro* studies on anti-inflammatory and immune-modulatory activity^32^, anti-proliferative effects in colon cancer cells^33^, and *in vivo* anti-inflammatory effects in animal model^34^ strengthen the therapeutic applicability. In the present clinical trial, we demonstrated that *B*. *coagulans* Unique IS2 was efficacious in restoration of CSBM, reduction of abdominal pain and other IBS associated symptoms (bloating, incomplete evacuation, urgency, straining, passage of gas, bowel habit satisfaction, and stool consistency) in adults (18–60 years).

In IBS patients, it has been observed that altered intestinal microbiota is commonly linked with changes in gastrointestinal function^1,35^. Probiotic mediated gut microbiota modulation is well documented to relieve symptoms of IBS^12^. Until now, several probiotic strains claim their efficacy in the treatment of IBS^12^. However, efficacy of the genus *Bacillus* has been scantily reported. Abdominal discomfort or pain and unsatisfactory bowel evacuations are the primary concerns for patients with IBS. The study conducted by Hun reported that patients (23–70 years) supplemented daily with 0.8 billion CFU of *B*. *coagulans* (GBI-30, 6086) for 8 weeks reduced baseline pain intensity score from 1.79 to 1.39 (*n* = *22*) as compared to placebo (1.43 to 1.16; *n* = *22*)^18^. Similarly, the supplementation of *B*. *coagulans* (0.15 billion CFU) + fructo-oligosaccharides (100 mg), 3 times a day for 12 weeks in adults (~39.8 years) reduced baseline pain from 6.0 to 1.8 (*n* = *23*) as compared to placebo (6.6 to 4.6; *n* = *33*)^36^. In this study, *B*. *coagulans* Unique IS2 (2 billion CFU/capsule/day) treatment for 8 weeks reduced baseline pain from 8.2 to 2.8 (*n* = *53*) as compared to placebo (8.3 to 7.0; *n* = *55*) in adults (18–60 years). These results are significant and have the advantage of a large number of participants in the trial. Moreover, at the end of trial, 90.57% patients with *B*. *coagulans* IS2 showed more than 50% pain reduction.

According to Food and Drug Administration (FDA), IBS-constipated trial responders are the patients who report, (i) ≥30% improvement in the daily average worst abdominal pain score from the baseline, and (ii) increase of ≥1 CSBM from the baseline, during the week, at least 50% of the weeks of treatment (6 of 12 weeks)^37^. In this study, *B*. *coagulans* treatment showed ≥50% improvement in abdominal pain score and increase of CSBM from baseline, which is in agreement to FDA. The mean CSBM score with *B*. *coagulans* was significantly increased from 2.5 to 4.0 with 42 (79.25%; *n* = *53*) patients experiencing CSBM at the end of treatment. Similarly, Kim and co-workers^38^ have shown that a 2 week/twice daily supplementation of VSL#3 (*Bifidobacterium breve*, *B*. *infantis*, *B*. *longum*, *Lactobacillus acidophilus*, *L*. *bulgaricus*, *L*. *casei*, *L*. *plantarum*, and *Streptococcus thermophilus*) probiotic significantly improved baseline CSBM score from 2.5 to 6.3 in patients with functional constipation (*n* = *30*; 20–59 years).

The total symptoms severity score is one of the most important determinants to demonstrate efficacy of therapeutic agents in IBS. The present clinical trial showed that mean score of total symptoms severity was significantly decreased from 26.7 to 10.6 as compared to placebo (26.7 to 21.5) during 8 weeks of *B*. *coagulans* Unique IS2 treatment. The percent total symptoms severity score difference between baseline and *B*. *coagulans* Unique IS2 treatment group at week 8 was 60%, which is higher as compared to 46% reported by Williams and co-workers^39^ for multi-strain probiotic (*L*. *acidophilus* NCIMB 30157, 30156, *B*. *bifidum* NCIMB 30153 and *B*. *lactis* NCIMB 30172).

The majority of patients treated with *B*. *coagulans* Unique IS2 achieved normal stool consistency as compared to placebo, regardless of the separate differentiation of study population for IBS subtypes. The study of Yoon and co-workers^40^ have shown that daily supplementation of LacClean Gold-S^®^ (*L*. *acidophilus*, *L*. *rhamnosus*, *S*. *thermophilus*, *B*. *bifidum*, *B*. *lactis*, and *B*. *longum*) was not effective to achieve normal consistency in patients with undetermined IBS subtypes. Overall, these results indicated superiority and efficacy of *B*. *coagulans* in stool consistency improvement in IBS patients. Furthermore, the administration of *B*. *coagulans* Unique IS2 resulted in improved scores of Physician’s and Patient’s global assessment (complete and considerable relief from IBS symptoms).

Immune system activation by enteric infections and associated inflammation aggravate IBS symptoms in individuals with psychosocial and genetic inclination^41^. A few studies have reported the changes in pro- (IL-6, IL-12, TNF-α, INF- γ) and anti- (IL-10) inflammatory cytokine levels in serum of IBS patients^41,42^. However, due to contradictory results, the serum cytokine profiling, of IBS patients remains indecisive. In the present investigation, eight week supplementation of *B*. *coagulans* Unique IS2 failed to show significant changes in the levels of pro- and anti-inflammatory serum cytokines (IL-6, IL-10, IL-12, TNF-α, INF- γ) as compared with placebo. These results are in agreement to the previous studies^43,44^, that the role of serum cytokines in the pathophysiology of IBS being unclear.

The safety of probiotic *B*. *coagulans* Unique IS2 has been already established and reported^22,24–33^. In this study, no severe adverse events and deaths with probiotic treatment were detected, which suggested safety of *B*. *coagulans* Unique IS2. Furthermore, the results on vital signs, physical examinations, hematology and usage of rescue medications during probiotic treatment support the safety and tolerance of *B*. *coagulans*.

In conclusion, as probiotic effects are strain specific, *B*. *coagulans* Unique IS2 significantly reduced abdominal pain and increased number of CSBM as compared to placebo in IBS patients Symptoms severity domains comprising bloating, incomplete evacuation, urgency, straining, passage of gas, bowel habit satisfaction, and stool consistency improved from baseline with *B*. *coagulans* treatment as compared to placebo. The decreased usage of rescue medications and few adverse events in the *B*. *coagulans* treated group compared with placebo further establish the efficacy and safety of *B*. *coagulans* Unique IS2 in IBS in adults.

## Methods

### Ethics and informed consent

This multicentric study was conducted at the Nanal Clinic and Life Veda Treatment and Research Centre, Mumbai, India. The trial was registered on 31/07/2017 with Clinical Trial Registry, India (CTRI/2017/07/009170; http://ctri.nic.in) and approved by Intersystem Biomedica Ethics Committee (ISBEC/NR-06/KM-VM/2016; IHS/UBL/01/16; 29/03/2016) and executed as per the guidelines of Declaration of Helsinki and ICMR, India. Patient signed informed consent forms were obtained by giving detailed oral and written information including risk and benefits associated with the current trial. The trial details can be accessed athttp://ctri.nic.in/Clinicaltrials/showallp.php?mid1 = 15233&EncHid = &userName = CTRI/2017/07/009170.

### Study design and selection of patients

This was a randomized, double blind, placebo controlled trial design with 2 week single blind or open-label screening/run-in period. The randomization was done in 1:1 ratio and generated by statistical analysis system (SAS) version 9.4. It consisted of four phases: screening, baseline visit (week 0 ± 5 days), visit 1 (week 4 ± 5 day), visit 2 (week 8 ± 5 days) and visit 3 (week 10 ± 5 days, telephonic follow-up) (Table S8).

A total of 153 patients (male/female, 18–60 years) with IBS, fulfilling Rome III criteria^2^ i.e. abdominal discomfort/pain associated with two or more of the following at least 25% of the time: improvement with defecation, onset associated with change in frequency of stool/and or in the form (appearance) of stool were supplemented with placebo during a 2 week screening period. Moreover, physical examination, vital signs (pulse rate, respiratory rate, blood pressure, and temperature), complete medical history and medications were assessed.

After establishing the eligibility on screening (80% compliance to the presence and persistence of trial entry criterion), patients were called for baseline visit (randomisation/day 0). The randomization was done on the basis of qualification to inclusion and exclusion criteria. The investigation product (*B*. *coagulans* Unique IS2, 2 billion CFU/capsule) or placebo (identical in size and appearance to the probiotic capsule but contained only excipient, maltodextrin) was administered to qualified patients for up to 8 weeks, followed by observation and telephonic follow-up (Table S8). The complete blood count, serum creatinine, serum glutamate pyruvate transaminase (SGPT), and the levels of serum cytokines (pro-inflammatory IL-6, 12, TNF-α, INF-γ and anti-inflammatory IL-10) were measured. Physical and vital examinations were recorded. Diaries were provided to patients for the assessment of pain logs for complete spontaneous bowel movement (CSBM), severity of symptoms (abdominal pain/discomfort, bloating/distension, urgency, incomplete evacuation, straining, passage of gas, bowel habit satisfaction, overall assessment of IBS symptoms, and change in severity of symptoms) and stool consistency. Adverse events, if any were documented and usage of any rescue or concomitant medication was reviewed and documented.

#### Inclusion criteria

(a) patients of either sex in the age group of 18–60 years, (b) fulfilling Rome III criteria of IBS, (c) no evidence of inflammatory, anatomic and metabolic or neoplastic process, (d) weekly, average worst abdominal pain score of ≥3.0 on 11 point scale, (e) average of less than 3 CSBMs per week (not due to the laxatives), and (f) able to provide informed consent.

#### Exclusion criteria

(a) patients with Bristol stool scale score of 7 or 6 for >25% of their bowel movements during the 12 weeks before screening or, during the run-in period (except laxative induce effect). (b) disease that may affect bowel motility other than IBS, (c) presence of rectal bleeding, recent weight loss (>5 kg in the past month) or iron deficiency anemia, (d) history of lactose intolerance and other malabsorption syndromes (*e*.*g*. fructose), (e) previous abdominal surgery and severe systemic diseases, (f) use of probiotic within 3 months of screening visit, (g) pregnant or breast-feeding or planning on becoming pregnant/women of child-bearing potential not using effective contraception, (h) use of any antibiotics (*e*.*g*. neomycin, rifaximin) within 1 month of screening, (i) daily use of laxative within one month of screening/current usage, or usage from the past 3 months, of narcotics or other medications for IBS management (*e*.*g*. alosetron, tegaserod and lubiprostone).

#### Discontinuation criteria

(a) patients were free to drop out from the study at any time without stating any reason (b) investigator could withdraw the patients from the study at any time due to adverse event or laboratory abnormality, non-compliance to visit requirements per protocol (±5 days), <80% drug compliance, intake of any prohibited medications, pregnancy, repeated and frequent non-adherence to prescribed dosing regimen (window period of 7 days), and worsening of condition or disorder.

### Sample size determination

Sample size was calculated using SAS software. The following assumption was made to detect presence of proportion difference. A minimum of 134 patients were required to be screened and 98 patients required evaluating the primary endpoint, which will provide 80% power to reject the null hypothesis (H0 = Test (%) – Placebo (%) = 0 verses Ha = Test (%) – Placebo (%) ≠ 0) when the true overall response is minimum 30% at a significant level of 0.05.

### Randomization, treatment allocation and procedures

Patients were randomized into two treatment arms (*B*. *coagulans* Unique IS2 and placebo) according to standard operating procedures. The study medications (*B*. *coagulans* Unique IS2 and matching placebo) and randomization code were kept blinded. The patients were instructed to consume one capsule post meal (as per the randomization schedule) per day for 8 weeks. During the trial, patients were prohibited to take medications/therapies (Table S9). Rescue medications that could be taken in case a need arose (mild infections or allergies) were listed (Table S10). The blinded information (in sealed envelopes) was supplied to each sites in case of emergency. The compliance during the run-in phase was maintained to 80%.

### Efficacy and safety measurement criteria

The primary efficacy outcomes were measured by assessing, (a) pain intensity on 11-point numerical rating scale (NRS)^44^ and (b) frequency of CSBM/SBM. The secondary efficacy outcomes were measured by (a) severity of symptoms on 6-point Likert scale^45^, (b) stool consistency on Bristol stool scale^46^, (c) patient and physician global assessment^47,48^, and (d) serum biomarker (TNF α, γ, and IL 6, 10, 12) levels (pictograms/ml) using ELISA (Diaclone Research, France). The safety was assessed by adverse event reporting, physical examination, monitoring of vital signs (heart rate, respiratory rate, blood pressure and temperature) and laboratory investigations (complete blood count).

### Statistical analysis

The SAS software (SAS^®^, Version 9.4, USA) was used for statistical evaluations. Chi-square/two sample *t-*test was performed to calculate statistical significance for pain intensity, CSBM, serum cytokine profile and severity of symptoms. Physician and patient global assessment was evaluated as frequency distribution and significance was assessed with chi-square test. A *p* value < 0.05 was considered as statistically significant.

## Supplementary information

Supplementary Info

## Data Availability

All data is available as a main text and supplementary material.

## References

[1] Stern EK, Brenner DM (2018). Gut microbiota-based therapies for irritable bowel syndrome. Clin. Transl. Gastroenterol..

[2] Lacy BE, Patel NK (2017). Rome criteria and a diagnostic approach to irritable bowel syndrome. J. Clin. Med..

[3] Lin LD, Chang L (2018). Using the Rome IV criteria to help manage the complex IBS patient. Am. J. Gastroenterol..

[4] Allergan. IBS global impact report 2018, https://www.badgut.org/wp-content/uploads/IBS-Global-Impact-Report.pdf. Accessed June 11, (2018).

[5] Canavan C, West J, Card T (2014). The epidemiology of irritable bowel syndrome. Clin. Epidemiol..

[6] Rodiño-Janeiro BK (2018). A review of microbiota and irritable bowel syndrome: future in therapies. Adv. Ther..

[7] Barbara G (2016). The intestinal microenvironment and functional gastrointestinal disorders. Gastroenterology.

[8] Saha L (2014). Irritable bowel syndrome: pathogenesis, diagnosis, treatment, and evidence-based medicine. World J. Gastroenterol..

[9] Occhipinti K, Smith JW (2012). Irritable bowel syndrome: a review and update. Clin. Colon Rectal Surg..

[10] Peyton L, Greene J (2014). Irritable bowel syndrome: current and emerging treatment options. Pharm. Ther..

[11] FAO/WHO. Guidelines for the evaluation of probiotics in food, report of a joint FAO/WHO working group on drafting guidelines for the evaluation of probiotics in food 2002, http://www.who.int/foodsafety/fs_management/en/probiotic_guidelines.pdf. Accessed June 11, (2018).

[12] Hungin AP (2018). Systematic review: probiotics in the management of lower gastrointestinal symptoms–an updated evidence‐based international consensus. Aliment. Pharmacol. Ther..

[13] Zhang Y (2016). Effects of probiotic type, dose and treatment duration on irritable bowel syndrome diagnosed by Rome III criteria: a meta-analysis. BMC Gastroenterol..

[14] Patel AK (2009). Comparative accounts of probiotic characteristics of *Bacillus* spp. isolated from food wastes. Food Res. Int..

[15] Ahire JJ (2011). *Bacillus* spp. of human origin: a potential siderophoregenic probiotic bacteria. Appl. Biochem. Biotechnol..

[16] Elshaghabee FMF (2017). *Bacillus* as potential probiotics: status, concerns, and future perspectives. Front. Microbiol..

[17] Scarpellini E (2006). *Bacillus clausii* treatment of small intestinal bacterial overgrowth in patients with irritable bowel syndrome. Dig. Liver. Dis..

[18] Hun L (2009). *Bacillus coagulans* significantly improved abdominal pain and bloating in patients with IBS. Postgrad. Med..

[19] Dolin BJ (2009). Effects of a proprietary *Bacillus coagulans* preparation on symptoms of diarrhea-predominant irritable bowel syndrome. Methods Find Exp. Clin. Pharmacol..

[20] Cobb, M. L. & Cobb, A. Inventors; Cobb and Co LLP, assignee. Treatment of irritable bowel syndrome using probiotic composition. United States patent US 7731976 (2010).

[21] Majeed M (2015). *Bacillus coagulans* MTCC 5856 supplementation in the management of diarrhea predominant irritable bowel syndrome: a double blind randomized placebo controlled pilot clinical study. Nutr. J..

[22] Sudha MR (2018). Efficacy of *Bacillus coagulans* Unique IS2 in treatment of irritable bowel syndrome in children: a double blind, randomised placebo controlled study. Benef. Microbes.

[23] Sudha R (2010). Molecular typing and probiotic attributes of a new strain of *Bacillus coagulans* Unique IS-2: a potential biotherapeutic agent. Genet. Eng. Biotechnol. J..

[24] Sudha RM, Sunita M, Sekhar BM (2016). Safety studies of *Bacillus coagulans* Unique IS-2 in rats: morphological, biochemical and clinical evaluations. Int. J. Probiotics Prebiotics.

[25] Sudha MR, Radkar N, Maurya A (2011). Effect of supplementation of probiotic *Bacillus coagulans* Unique IS-2 (ATCC PAT-11748) on hypercholesterolemic subjects: a clinical study. Int. J. Probiotics Prebiotics.

[26] Pawar RR, Pardeshi ML, Ghongane BB (2012). Study of effects of probiotic lactobacilli in preventing major complications in patients of liver cirrhosis. Int. J. Res. Pharma. Biomed. Sci..

[27] Sudha RM, Yelikar KA, Deshpande S (2012). Clinical study of *Bacillus coagulans* Unique IS-2 (ATCC PTA-11748) in the treatment of patients with bacterial vaginosis. Indian J. Microbiol..

[28] Sudha RM, Bhonagiri S (2012). Efficacy of *Bacillus coagulans* strain Unique IS-2 in the treatment of patients with acute diarrhea. Int. J. Probiotics Prebiotics.

[29] Saneian H (2015). Synbiotic containing *Bacillus coagulans* and fructo-oligosaccharides for functional abdominal pain in children. Gastroenterol. Hepatol. Bed Bench.

[30] Madempudi, R. S. *et al*. *Bacillus coagulans* Unique IS2 in constipation: a double-blind, placebo-controlled study. *Probiotics Antimicrob*. *Proteins*, 10.1007/s12602-019-09542-9 (2019). 10.1007/s12602-019-09542-9 30911991

[31] Maithri JK (2017). Clinical effect of probiotic containing *Bacillus coagulans* on plaque induced gingivitis: a randomised clinical pilot study. Nitte. Uni. J. Health Sci..

[32] Sudha MR, Arunasree KM (2015). Anti-inflammatory and immunomodulatory effects of *Bacillus coagulans* Unique IS2. Int. J. Probiotics Prebiotics.

[33] Madempudi RS, Kalle AM (2017). Anti-proliferative effects of *Bacillus coagulans* Unique IS2 in colon cancer cells. Nutr. Cancer.

[34] Solanki HK (2015). Evaluation of anti-inflammatory activity of probiotic on carrageenan-induced paw edema in Wistar rats. Int. J. Biol. Macromol..

[35] Ringel Y, Maharshak N (2013). Intestinal microbiota and immune function in the pathogenesis of irritable bowel syndrome. Am. J. Physiol. Gastrointest. Liver. Physiol..

[36] Rogha M, Esfahani MZ, Zargarzadeh AH (2014). The efficacy of a synbiotic containing *Bacillus coagulans* in treatment of irritable bowel syndrome: a randomized placebo-controlled trial. Gastroenterol. Hepatol. Bed Bench.

[37] Macdougall JE (2013). An evaluation of the FDA responder endpoint for IBS‐C clinical trials: analysis of data from linaclotide phase 3 clinical trials. Neurogastroenterol. Motil..

[38] Kim SE (2015). Change of fecal flora and effectiveness of the short-term VSL# 3 probiotic treatment in patients with functional constipation. Neurogastroenterol. Motil..

[39] Williams EA (2009). Clinical trial: a multistrain probiotic preparation significantly reduces symptoms of irritable bowel syndrome in a double‐blind placebo‐controlled study. Aliment. Pharmacol. Ther..

[40] Yoon JS (2014). Effect of multispecies probiotics on irritable bowel syndrome: A randomized, double‐blind, placebo‐controlled trial. J. Gastroenterol. Hepatol..

[41] Hua MC (2011). Decreased interleukin-10 secretion by peripheral blood mononuclear cells in children with irritable bowel syndrome. J. Pediatr. Gastroenterol. Nutr..

[42] Bennet SM (2016). Global cytokine profiles and association with clinical characteristics in patients with irritable bowel syndrome. Am. J. Gastroenterol..

[43] Svensson CI (2010). Interleukin-6: a local pain trigger?. Arthritis Res. Ther..

[44] Lazaridis N, Germanidis G (2018). Current insights into the innate immune system dysfunction in irritable bowel syndrome. Ann. Gastroenterol..

[45] Mujagic Z (2015). Systematic review: instruments to assess abdominal pain in irritable bowel syndrome. Aliment. Pharmacol. Ther..

[46] Blake MR, Raker JM, Whelan K (2016). Validity and reliability of the Bristol stool form scale in healthy adults and patients with diarrhoea‐predominant irritable bowel syndrome. Aliment. Pharmacol. Ther..

[47] Müller-Lissner S (2003). Subject’s global assessment of relief: an appropriate method to assess the impact of treatment on irritable bowel syndrome-related symptoms in clinical trials. J. Clin. Epidemiol..

[48] Halpert A (2018). Irritable bowel syndrome: patient-provider interaction and patient education. J. Clin. Med..

