# Supplementary material for: Randomized clinical trial: the effect of probiotic Bacillus coagulans Unique IS2 vs. placebo on the symptoms management of irritable bowel syndrome in adults
Source: Sci Rep. 2019 Aug 21;9:12210. doi: 10.1038/s41598-019-48554-x (PMC6704184; doi:10.1038/s41598-019-48554-x)
Supplement: Supplementary file 1 — Supplementary Info [file 41598_2019_48554_MOESM1_ESM.docx]

Randomized clinical trial: the effect of probiotic *Bacillus coagulans* Unique IS2 vs. placebo on the symptoms management of irritable bowel syndrome in adults.

Ratna Sudha Madempudi^1,^*, Jayesh J. Ahire^1^, Jayanthi Neelamraju^1^, Anirudh Tripathi^2^, Satyavrat Nanal^3^

^1^Centre for Research & Development, Unique Biotech Ltd., Plot No. 2, Phase-II, Alexandria Knowledge Park, Hyderabad, Telangana 500078, India.

^2^Life Veda Treatment and Research Centre, Worli, Mumbai 400030, India.

^3^Nanal Clinic, Anand Bhuvan, Gore wadi, Mahim (W), Mumbai 400016, India

*Correspondence to:

Dr. Ratna Sudha Madempudi, Unique Biotech Ltd., Hyderabad, India.

Email: sudha@uniquebiotech.com

**Supplementary Tables**

**Table S1.** Patients baseline disease characteristics.

|  | *B. coagulans*  (*n = 53*) | Placebo  (*n = 55*) | Total (*n = 108*) | *p* value |
| --- | --- | --- | --- | --- |
| (I) IBS subtype by predominant bowel habit: *n* (%) | | | | |
| Diarrhea (IBS-D) | 0 | 3 (5.45) | 3 (2.78) | 0.0846* |
| Constipation (IBS-C) | 18 (33.96) | 17 (30.91) | 35 (32.41) | 0.7347* |
| Mixed (IBS-M) | 35 (66.04) | 35 (63.64) | 70 (64.81) | 0.7939* |
| (II) CSBM | | | | |
| Mean CSBM score | 2.5 | 2.2 | 2.3 | 0.2825 |
| (III) Abdominal discomfort | | | | |
| Mean abdominal discomfort Scores | 3.6 | 3.6 | 3.6 | 0.5306 |

*p* value: inter group (chi-square* and two sample *t* test).

**Table S2.** Patients medical and disease history classified by medical dictionary for regulatory activities (MedDRA).

| SOC | Preferred term | *B. coagulans* (*n = 53*) | | Placebo  (*n = 55*) | | Total  (*n = 108*) | |
| --- | --- | --- | --- | --- | --- | --- | --- |
|  |  | *n* | % | *n* | % | *n* | % |
| GID | Abdominal bloating and pain with abnormal changes in stool frequency. | 2 | 3.77 | 1 | 1.82 | 3 | 2.78 |
| GID | Abdominal discomfort and abdominal pain. | 1 | 1.89 | 0 | 0 | 1 | 0.93 |
| GID | Abdominal pain and bloating with change in stool form. | 4 | 7.55 | 2 | 3.64 | 6 | 5.56 |
| GID | Abdominal pain and bloating. | 6 | 11.32 | 2 | 3.64 | 8 | 7.41 |
| GID | Abdominal pain and incomplete evacuation. | 0 | 0 | 1 | 1.82 | 1 | 0.93 |
| GID | Abdominal pain and passage of gas. | 3 | 5.66 | 1 | 1.82 | 4 | 3.70 |
| GID | Abdominal pain and straining. | 1 | 1.89 | 1 | 1.82 | 2 | 1.85 |
| GID | Abdominal pain with change in stool form. | 10 | 18.87 | 4 | 7.27 | 14 | 12.96 |
| GID | Abdominal pain with change in stool frequency and form of stool. | 1 | 1.89 | 3 | 5.45 | 4 | 3.70 |
| GID | Abdominal pain with change in stool frequency. | 5 | 9.43 | 8 | 14.55 | 13 | 12.04 |
| GID | Abdominal pain. | 4 | 7.55 | 8 | 14.55 | 12 | 11.11 |
| GID | Abnormal form of stool with abdominal pain and straining. | 1 | 1.89 | 2 | 3.64 | 3 | 2.78 |
| GID | Abnormal stool form and frequency with abdominal pain and bloating. | 1 | 1.89 | 2 | 3.64 | 3 | 2.78 |
| GID | Abnormal stool form with abdominal pain and incomplete evacuation. | 1 | 1.89 | 1 | 1.82 | 2 | 1.85 |
| GID | Abnormal stool form with abdominal pain and straining. | 0 | 0 | 1 | 1.82 | 1 | 0.93 |
| GID | Abnormal stool form with abdominal pain, bloating and passage of gas. | 1 | 1.89 | 0 | 0 | 1 | 0.93 |
| GID | Abnormal stool form with bloating. | 2 | 3.77 | 2 | 3.64 | 4 | 3.70 |
| GID | Abnormal stool frequency and bloating and discomfort. | 1 | 1.89 | 0 | 0 | 1 | 0.93 |
| GID | Abnormal stool frequency with abdominal pain, bloating and gas | 0 | 0 | 1 | 1.82 | 1 | 0.93 |
| GID | Bloating and abdominal pain with change in stool form. | 0 | 0 | 2 | 3.64 | 2 | 1.85 |
| GID | Bloating and pain with abnormal stool form. | 1 | 1.89 | 1 | 1.82 | 2 | 1.85 |
| GID | Bloating with change in stool form. | 2 | 3.77 | 1 | 1.82 | 3 | 2.78 |
| GID | Bloating with change in stool frequency and form. | 0 | 0 | 3 | 5.45 | 3 | 2.78 |
| GID | Bloating. | 3 | 5.66 | 5 | 9.09 | 8 | 7.41 |
| GID | Change in stool form and frequency with straining and abdominal pain. | 1 | 1.89 | 0 | 0 | 1 | 0.93 |
| GID | (Heartburn) acidity | 1 | 1.89 | 0 | 0 | 1 | 0.93 |
| MCTD | Back pain | 2 | 3.77 | 0 | 0 | 2 | 1.85 |
| GID | Constipation | 1 | 1.89 | 2 | 3.64 | 3 | 2.87 |
| MND | Diabetes mellitus | 0 | 0 | 1 | 1.82 | 1 | 0.93 |
| GID | Hyperacidity | 1 | 1.89 | 0 | 0 | 1 | 0.93 |
| VD | Hypertension | 2 | 3.77 | 0 | 0 | 2 | 1.85 |
| HD | Jaundice | 1 | 1.89 | 2 | 3.64 | 3 | 2.78 |
| MCTD | Arthralgia | 1 | 1.89 | 1 | 1.82 | 2 | 1.85 |
| BLSD | Malaria | 0 | 0 | 1 | 1.82 | 1 | 0.93 |
| MCTD | Shoulder pain | 1 | 1.89 | 0 | 0 | 1 | 0.93 |

*n*, %: Number and percentage of patients with given characteristics. SOC: System organ class. GID: Gastrointestinal disorder. MCTD: Musculoskeletal and connective tissue disorders. MND: Metabolism and nutrition disorders. VD: Vascular disorders. HD: Hepatobiliary disorders. BLSD: Blood and lymphatic system disorder.

**Table S3.** Percentage of patients who used prior medications (1–3 months before inclusion in study).

| Preferred term | *B. coagulans* (*n = 53*) | | Placebo (*n = 55*) | | Total (*n = 108*) | |
| --- | --- | --- | --- | --- | --- | --- |
|  | *n* | % | *n* | % | *n* | % |
| Duphalac syrup (lactulose) | 0 | 0 | 2 | 3.64 | 2 | 1.85 |
| Lomotil syrup (diphenoxylate and atropine) | 1 | 1.89 | 0 | 0 | 1 | 0.93 |
| Relux syrup (polyethylene glycol) | 1 | 1.89 | 0 | 0 | 1 | 0.93 |
| Tab. Bi-Quinol (*Aegle marmelous, Hollerhena antidysentrica, Bombax malbaricum, Hollerhena antidysentrica* seeds and *Berberine*) | 1 | 1.89 | 0 | 0 | 1 | 0.93 |
| Tab. Consti-go (lactulose) | 1 | 1.89 | 0 | 0 | 1 | 0.93 |
| Tab. Furazole-M (metrodizazole and furazolidone) | 0 | 0 | 1 | 1.82 | 1 | 0.93 |
| Tab. Laxomed (*Cassia fistula*, *Curcuma angustifolia* and *Glycyrrhiza glabra*) | 0 | 0 | 2 | 3.64 | 2 | 1.85 |
| Tab. Lomofen (atropine diphenoxylate and furazolidone) | 0 | 0 | 1 | 1.82 | 1 | 0.93 |
| Tab. Metrogyl 400 (metronidazole) | 1 | 1.89 | 0 | 0 | 1 | 0.93 |
| Tab. Consti-go (lactulose) | 1 | 1.89 | 0 | 0 | 1 | 0.93 |
| Tab. Constipeg (polyethylene glycol) | 0 | 0 | 1 | 1.82 | 1 | 0.93 |

*n*, %: Number and percentage of patients with given characteristics.

**Table S4.** Analysis of absolute change in severity of symptoms domain as evaluated by IBS severity scoring system.

| Visit | *B. coagulans* | | | Placebo | | |  | | | | *p* value** | |
| --- | --- | --- | --- | --- | --- | --- | --- | --- | --- | --- | --- | --- |
|  | *n* | Mean | SD | *n* | Mean | SD | Mean | SD | CI | *p* value* | *B. coagulans* | Placebo |
| Abdominal discomfort | | | | | | | | | | | | |
| Baseline | 53 | 3.6 | 0.60 | 55 | 3.6 | 0.56 |  |  |  |  |  |  |
| Week 4 | 53 | 2.6 | 0.68 | 55 | 3.1 | 0.69 | -0.7 | 0.8 | (-0.87,-0.57) | 0.0088 | <.0001 | <.0001 |
| Week 08 | 53 | 1.3 | 0.85 | 55 | 2.8 | 0.87 | -1.5 | 1.2 | (-1.77,-1.30) | <.0001 | <.0001 | <.0001 |
| Bloating/distension | | | | | | | | | | | | |
| Baseline | 53 | 3.4 | 0.75 | 55 | 3.3 | 0.58 |  |  |  |  |  |  |
| Week 4 | 53 | 2.5 | 0.77 | 55 | 2.9 | 0.69 | -0.7 | 0.9 | (-0.82,-0.49) | 0.0063 | <.0001 | 0.0005 |
| Week 08 | 53 | 1.4 | 0.80 | 55 | 2.7 | 1.00 | -1.3 | 1.3 | (-1.55,-1.06) | <.0001 | <.0001 | <.0001 |
| Urgency | | | | | | | | | | | | |
| Baseline | 53 | 3.0 | 0.48 | 55 | 3.1 | 0.57 |  |  |  |  |  |  |
| Week 4 | 53 | 2.3 | 0.72 | 55 | 2.8 | 0.76 | -0.5 | 0.8 | (-0.63,-0.31) | 0.0025 | <.0001 | 0.0694 |
| Week 08 | 53 | 1.3 | 0.89 | 55 | 2.5 | 1.00 | -1.1 | 1.2 | (-1.35,-0.89) | <.0001 | <.0001 | 0.0005 |
| Incomplete evacuation | | | | | | | | | | | | |
| Baseline | 53 | 3.1 | 0.36 | 55 | 3.2 | 0.45 |  |  |  |  |  |  |
| Week 4 | 53 | 2.3 | 0.70 | 55 | 2.6 | 0.65 | -0.7 | 0.7 | (-0.80,-0.54) | 0.1902 | <.0001 | <.0001 |
| Week 08 | 53 | 1.2 | 0.76 | 55 | 2.5 | 0.90 | -1.3 | 1.0 | (-1.46,-1.06) | <.0001 | <.0001 | <.0001 |
| Straining | | | | | | | | | | | | |
| Baseline | 53 | 3.2 | 0.49 | 55 | 3.2 | 0.51 |  |  |  |  |  |  |
| Week 4 | 53 | 2.2 | 0.81 | 55 | 2.6 | 0.71 | -0.8 | 0.8 | (-0.93,-0.61) | 0.0193 | <.0001 | <.0001 |
| Week 08 | 53 | 1.1 | 0.65 | 55 | 2.5 | 0.98 | -1.3 | 1.1 | (-1.56,-1.13) | <.0001 | <.0001 | <.0001 |
| Passage of gas | | | | | | | | | | | | |
| Baseline | 53 | 3.2 | 0.60 | 55 | 3.3 | 0.53 |  |  |  |  |  |  |
| Week 4 | 53 | 2.2 | 0.78 | 55 | 2.7 | 0.79 | -0.8 | 1.0 | (-0.98,-0.59) | 0.0505 | <.0001 | <.0001 |
| Week 08 | 53 | 1.2 | 0.76 | 55 | 2.6 | 0.85 | -1.3 | 1.3 | (-1.58,-1.10) | <.0001 | <.0001 | <.0001 |
| Bowel habit satisfaction | | | | | | | | | | | | |
| Baseline | 53 | 3.3 | 0.63 | 55 | 3.4 | 0.63 |  |  |  |  |  |  |
| Week 4 | 53 | 2.5 | 0.82 | 55 | 3.0 | 0.69 | -0.6 | 0.9 | (-0.77,-0.41) | 0.0809 | <.0001 | 0.0008 |
| Week 08 | 53 | 1.4 | 0.74 | 55 | 2.9 | 0.87 | -1.2 | 1.2 | (-1.39,-0.93) | <.0001 | <.0001 | 0.0022 |
| Overall assessment | | | | | | | | | | | | |
| Baseline | 53 | 3.6 | 0.57 | 55 | 3.6 | 0.53 |  |  |  |  |  |  |
| Week 4 | 53 | 2.8 | 0.90 | 55 | 3.2 | 0.72 | -0.6 | 0.9 | (-0.75,-0.42) | 0.0119 | <.0001 | 0.0020 |
| Week 08 | 53 | 1.6 | 0.86 | 55 | 3.0 | 0.83 | -1.3 | 1.1 | (-1.51,-1.08) | <.0001 | <.0001 | <.0001 |
| Total score | | | | | | | | | | | | |
| Baseline | 53 | 26.4 | 2.54 | 55 | 26.7 | 2.31 |  |  |  |  |  |  |
| Week 4 | 53 | 19.6 | 4.47 | 55 | 22.9 | 3.37 | -5.3 | 4.5 | (-6.10,-4.40) | 0.0004 | <.0001 | <.0001 |
| Week 08 | 53 | 10.6 | 5.26 | 55 | 21.5 | 5.88 | -10.4 | 7.9 | (-11.89,-8.85) | <.0001 | <.0001 | <.0001 |

*p**: inter group (two sample *t* test). *p***: intra group (two sample *t* test). *n*:number of patients.

**Table S5.** Summary of hematology.

|  | | *B. coagulans* (*n = 53*) | | | | | | Placebo (*n = 55*) | | | | | | Total (*n = 108*) | | | | | |
| --- | --- | --- | --- | --- | --- | --- | --- | --- | --- | --- | --- | --- | --- | --- | --- | --- | --- | --- | --- |
|  | | Normal | | Abnormal (CNS) | | Abnormal (CS) | | Normal | | Abnormal (CNS) | | Abnormal (CS) | | Normal | | Abnormal (CNS) | | Abnormal (CS) | |
| test | visit | *n* | % | *n* | % | *n* | % | *n* | % | *n* | % | *n* | % | *n* | % | *n* | % | *n* | % |
| RBC | Baseline | 51 | 96.23 | 2 | 3.77 | 0 | 0 | 52 | 94.55 | 3 | 5.45 | 0 | 0 | 103 | 95.37 | 5 | 4.63 | 0 | 0 |
|  | Week 8 | 49 | 92.45 | 4 | 7.55 | 0 | 0 | 52 | 94.55 | 3 | 5.45 | 0 | 0 | 101 | 93.52 | 7 | 6.48 | 0 | 0 |
| Haemoglobin | Baseline | 37 | 69.81 | 16 | 30.19 | 0 | 0 | 41 | 74.55 | 14 | 25.45 | 0 | 0 | 78 | 72.22 | 30 | 27.78 | 0 | 0 |
|  | Week 8 | 42 | 79.25 | 11 | 20.75 | 0 | 0 | 40 | 72.73 | 15 | 27.27 | 0 | 0 | 82 | 75.93 | 26 | 24.07 | 0 | 0 |
| MCV | Baseline | 40 | 75.47 | 13 | 24.53 | 0 | 0 | 48 | 87.27 | 7 | 12.73 | 0 | 0 | 88 | 81.48 | 20 | 18.52 | 0 | 0 |
|  | Week 8 | 37 | 69.81 | 16 | 30.19 | 0 | 0 | 47 | 85.45 | 8 | 14.55 | 0 | 0 | 84 | 77.78 | 24 | 22.22 | 0 | 0 |
| MCH | Baseline | 30 | 56.60 | 23 | 43.40 | 0 | 0 | 44 | 80.00 | 11 | 20.00 | 0 | 0 | 74 | 68.52 | 34 | 31.48 | 0 | 0 |
|  | Week 8 | 31 | 58.49 | 22 | 41.51 | 0 | 0 | 40 | 72.73 | 15 | 27.27 | 0 | 0 | 71 | 65.74 | 37 | 34.26 | 0 | 0 |
| MCHC | Baseline | 30 | 56.60 | 23 | 43.40 | 0 | 0 | 42 | 76.36 | 13 | 23.64 | 0 | 0 | 72 | 66.67 | 36 | 33.33 | 0 | 0 |
|  | Week 8 | 34 | 64.15 | 19 | 35.85 | 0 | 0 | 37 | 67.27 | 18 | 32.73 | 0 | 0 | 71 | 65.74 | 37 | 34.26 | 0 | 0 |
| WBC | Baseline | 48 | 90.57 | 5 | 9.43 | 0 | 0 | 51 | 92.73 | 4 | 7.27 | 0 | 0 | 99 | 91.67 | 9 | 8.33 | 0 | 0 |
|  | Week 8 | 51 | 96.23 | 2 | 3.77 | 0 | 0 | 53 | 96.36 | 2 | 3.64 | 0 | 0 | 104 | 96.30 | 4 | 3.70 | 0 | 0 |
| Neutrophils | Baseline | 41 | 77.36 | 12 | 22.64 | 0 | 0 | 46 | 83.64 | 9 | 16.36 | 0 | 0 | 87 | 80.56 | 21 | 19.44 | 0 | 0 |
|  | Week 8 | 47 | 88.68 | 6 | 11.32 | 0 | 0 | 44 | 80.00 | 11 | 20.00 | 0 | 0 | 91 | 84.26 | 17 | 15.74 | 0 | 0 |
| Basophils | Baseline | 51 | 96.23 | 2 | 3.77 | 0 | 0 | 51 | 92.73 | 4 | 7.27 | 0 | 0 | 102 | 94.44 | 6 | 5.56 | 0 | 0 |
|  | Week 8 | 52 | 98.11 | 1 | 1.89 | 0 | 0 | 53 | 96.36 | 2 | 3.64 | 0 | 0 | 105 | 97.22 | 3 | 2.78 | 0 | 0 |
| Eosinophils | Baseline | 47 | 88.68 | 6 | 11.32 | 0 | 0 | 47 | 85.45 | 8 | 14.55 | 0 | 0 | 94 | 87.04 | 14 | 12.96 | 0 | 0 |
|  | Week 8 | 46 | 86.79 | 7 | 13.21 | 0 | 0 | 51 | 92.73 | 4 | 7.27 | 0 | 0 | 97 | 89.81 | 11 | 10.19 | 0 | 0 |
| Lymphocytes | Baseline | 47 | 88.68 | 6 | 11.32 | 0 | 0 | 52 | 94.55 | 3 | 5.45 | 0 | 0 | 99 | 91.67 | 9 | 8.33 | 0 | 0 |
|  | Week 8 | 52 | 98.11 | 1 | 1.89 | 0 | 0 | 52 | 94.55 | 3 | 5.45 | 0 | 0 | 104 | 96.30 | 4 | 3.70 | 0 | 0 |
| Monocytes | Baseline | 34 | 64.15 | 19 | 35.85 | 0 | 0 | 39 | 70.91 | 16 | 29.09 | 0 | 0 | 73 | 67.59 | 35 | 32.41 | 0 | 0 |
|  | Week 8 | 35 | 66.04 | 18 | 33.96 | 0 | 0 | 38 | 69.09 | 17 | 30.91 | 0 | 0 | 73 | 67.59 | 35 | 32.41 | 0 | 0 |
| Platelet Count | Baseline | 44 | 83.02 | 9 | 16.98 | 0 | 0 | 52 | 94.55 | 3 | 5.45 | 0 | 0 | 96 | 88.89 | 12 | 11.11 | 0 | 0 |
|  | Week 8 | 47 | 88.68 | 6 | 11.32 | 0 | 0 | 50 | 90.91 | 5 | 9.09 | 0 | 0 | 97 | 89.81 | 11 | 10.19 | 0 | 0 |
| HCT | Baseline | 37 | 69.81 | 16 | 30.19 | 0 | 0 | 40 | 72.73 | 15 | 27.27 | 0 | 0 | 77 | 71.30 | 31 | 28.70 | 0 | 0 |
|  | Week 8 | 42 | 79.25 | 11 | 20.75 | 0 | 0 | 41 | 74.55 | 14 | 25.45 | 0 | 0 | 83 | 76.85 | 25 | 23.15 | 0 | 0 |
| Creatinine | Baseline | 52 | 98.11 | 1 | 1.89 | 0 | 0 | 53 | 96.36 | 2 | 3.64 | 0 | 0 | 105 | 97.22 | 3 | 2.78 | 0 | 0 |
|  | Week 8 | 53 | 100.00 | 0 | 0 | 0 | 0 | 54 | 98.18 | 1 | 1.82 | 0 | 0 | 107 | 99.07 | 1 | 0.93 | 0 | 0 |
| PCT | Baseline | 44 | 83.02 | 9 | 16.98 | 0 | 0 | 42 | 76.36 | 13 | 23.64 | 0 | 0 | 86 | 79.63 | 22 | 20.37 | 0 | 0 |
|  | Week 8 | 43 | 81.13 | 10 | 18.87 | 0 | 0 | 41 | 74.55 | 14 | 25.45 | 0 | 0 | 84 | 77.78 | 24 | 22.22 | 0 | 0 |
| SGPT | Baseline | 47 | 88.68 | 6 | 11.32 | 0 | 0 | 53 | 96.36 | 2 | 3.64 | 0 | 0 | 100 | 92.59 | 8 | 7.41 | 0 | 0 |
|  | Week 8 | 47 | 88.68 | 6 | 11.32 | 0 | 0 | 48 | 87.27 | 7 | 12.73 | 0 | 0 | 95 | 87.96 | 13 | 12.04 | 0 | 0 |

*n*/%: number/percentage of patients with given characteristics. RBC: red blood cells. MCV: mean corpuscular volume. MCH: mean corpuscular hemoglobin. MCHC: mean corpuscular hemoglobin concentration. WBC: white blood cells. HCT: hematocrit. PCT: procalcitonin. SGPT: serum glutamic pyruvic transaminase.

**Table S6.** Summary of physical examination.

|  | | *B. coagulans* (*n = 53*) | | | | | | Placebo (*n = 55*) | | | | | | Total (*n = 108*) | | | | | |
| --- | --- | --- | --- | --- | --- | --- | --- | --- | --- | --- | --- | --- | --- | --- | --- | --- | --- | --- | --- |
|  | | Normal | | Abnormal (CNS) | | Abnormal (CS) | | Normal | | Abnormal (CNS) | | Abnormal (CS) | | Normal | | Abnormal (CNS) | | Abnormal (CS) | |
| Test | Visit | *n* | % | *n* | % | *n* | % | *n* | % | *n* | % | *n* | % | *n* | % | *n* | % | *n* | % |
| General  Appearance | Screening | 53 | 100.00 | 0 | 0 | 0 | 0 | 55 | 100.00 | 0 | 0 | 0 | 0 | 108 | 100.00 | 0 | 0 | 0 | 0 |
|  | Baseline | 53 | 100.00 | 0 | 0 | 0 | 0 | 55 | 100.00 | 0 | 0 | 0 | 0 | 108 | 100.00 | 0 | 0 | 0 | 0 |
|  | Visit 1 | 53 | 100.00 | 0 | 0 | 0 | 0 | 55 | 100.00 | 0 | 0 | 0 | 0 | 108 | 100.00 | 0 | 0 | 0 | 0 |
|  | Visit 2 | 53 | 100.00 | 0 | 0 | 0 | 0 | 55 | 100.00 | 0 | 0 | 0 | 0 | 108 | 100.00 | 0 | 0 | 0 | 0 |
| Gastrointestinal  system | Screening | 0 | 0 | 53 | 100.00 | 0 | 0 | 0 | 0 | 55 | 100.00 | 0 | 0 | 0 | 0 | 108 | 100.00 | 0 | 0 |
|  | Baseline | 0 | 0 | 53 | 100.00 | 0 | 0 | 0 | 0 | 55 | 100.00 | 0 | 0 | 0 | 0 | 108 | 100.00 | 0 | 0 |
|  | Visit 1 | 2 | 3.77 | 51 | 96.23 | 0 | 0 | 0 | 0 | 55 | 100.00 | 0 | 0 | 2 | 1.85 | 106 | 98.15 | 0 | 0 |
|  | Visit 2 | 45 | 84.91 | 8 | 15.09 | 0 | 0 | 5 | 9.09 | 50 | 90.91 | 0 | 0 | 50 | 46.30 | 58 | 53.70 | 0 | 0 |
| Respiratory system | Screening | 53 | 100.00 | 0 | 0 | 0 | 0 | 55 | 100.00 | 0 | 0 | 0 | 0 | 108 | 100.00 | 0 | 0 | 0 | 0 |
|  | Baseline | 53 | 100.00 | 0 | 0 | 0 | 0 | 55 | 100.00 | 0 | 0 | 0 | 0 | 108 | 100.00 | 0 | 0 | 0 | 0 |
|  | Visit 1 | 53 | 100.00 | 0 | 0 | 0 | 0 | 55 | 100.00 | 0 | 0 | 0 | 0 | 108 | 100.00 | 0 | 0 | 0 | 0 |
|  | Visit 2 | 53 | 100.00 | 0 | 0 | 0 | 0 | 55 | 100.00 | 0 | 0 | 0 | 0 | 108 | 100.00 | 0 | 0 | 0 | 0 |
| Nervous  system | Screening | 53 | 100.00 | 0 | 0 | 0 | 0 | 55 | 100.00 | 0 | 0 | 0 | 0 | 108 | 100.00 | 0 | 0 | 0 | 0 |
|  | Baseline | 53 | 100.00 | 0 | 0 | 0 | 0 | 55 | 100.00 | 0 | 0 | 0 | 0 | 108 | 100.00 | 0 | 0 | 0 | 0 |
|  | Visit 1 | 53 | 100.00 | 0 | 0 | 0 | 0 | 55 | 100.00 | 0 | 0 | 0 | 0 | 108 | 100.00 | 0 | 0 | 0 | 0 |
|  | Visit 2 | 53 | 100.00 | 0 | 0 | 0 | 0 | 55 | 100.00 | 0 | 0 | 0 | 0 | 108 | 100.00 | 0 | 0 | 0 | 0 |
| Genitourinary system | Screening | 53 | 100.00 | 0 | 0 | 0 | 0 | 55 | 100.00 | 0 | 0 | 0 | 0 | 108 | 100.00 | 0 | 0 | 0 | 0 |
|  | Baseline | 53 | 100.00 | 0 | 0 | 0 | 0 | 55 | 100.00 | 0 | 0 | 0 | 0 | 108 | 100.00 | 0 | 0 | 0 | 0 |
|  | Visit 1 | 53 | 100.00 | 0 | 0 | 0 | 0 | 55 | 100.00 | 0 | 0 | 0 | 0 | 108 | 100.00 | 0 | 0 | 0 | 0 |
|  | Visit 2 | 53 | 100.00 | 0 | 0 | 0 | 0 | 55 | 100.00 | 0 | 0 | 0 | 0 | 108 | 100.00 | 0 | 0 | 0 | 0 |
| Musculo skeletal system | Screening | 51 | 96.23 | 2 | 3.77 | 0 | 0 | 52 | 94.55 | 3 | 5.45 | 0 | 0 | 103 | 95.37 | 5 | 4.63 | 0 | 0 |
|  | Baseline | 52 | 98.11 | 1 | 1.89 | 0 | 0 | 53 | 96.36 | 2 | 3.64 | 0 | 0 | 105 | 97.22 | 3 | 2.78 | 0 | 0 |
|  | Visit 1 | 52 | 98.11 | 1 | 1.89 | 0 | 0 | 53 | 96.36 | 2 | 3.64 | 0 | 0 | 105 | 97.22 | 3 | 2.78 | 0 | 0 |
|  | Visit 2 | 52 | 98.11 | 1 | 1.89 | 0 | 0 | 54 | 98.18 | 1 | 1.82 | 0 | 0 | 106 | 98.15 | 2 | 1.85 | 0 | 0 |
| Others | Screening | 53 | 100.00 | 0 | 0 | 0 | 0 | 55 | 100.00 | 0 | 0 | 0 | 0 | 108 | 100.00 | 0 | 0 | 0 | 0 |
|  | Baseline | 53 | 100.00 | 0 | 0 | 0 | 0 | 55 | 100.00 | 0 | 0 | 0 | 0 | 108 | 100.00 | 0 | 0 | 0 | 0 |
|  | Visit 1 | 53 | 100.00 | 0 | 0 | 0 | 0 | 55 | 100.00 | 0 | 0 | 0 | 0 | 108 | 100.00 | 0 | 0 | 0 | 0 |
|  | Visit 2 | 53 | 100.00 | 0 | 0 | 0 | 0 | 55 | 100.00 | 0 | 0 | 0 | 0 | 108 | 100.00 | 0 | 0 | 0 | 0 |

*n*/%: number/percentage of patients with given characteristics.

**Table S7.** Summary of vital signs.

|  | | | *B. coagulans* (*n = 53*) | | | | | | Placebo (*n = 55*) | | | | | | Total (*n = 108*) | | | | | |
| --- | --- | --- | --- | --- | --- | --- | --- | --- | --- | --- | --- | --- | --- | --- | --- | --- | --- | --- | --- | --- |
|  | | | Normal | | Abnormal (CNS) | | Abnormal (CS) | | Normal | | Abnormal (CNS) | | Abnormal (CS) | | Normal | | Abnormal (CNS) | | Abnormal (CS) | |
| Test |  | Visit | *n* | % | *n* | % | *n* | % | *n* | % | *n* | % | *n* | % | *n* | % | *n* | % | *n* | % |
| Pulse | | Screening | 53 | 100.00 | 0 | 0 | 0 | 0 | 55 | 100.00 | 0 | 0 | 0 | 0 | 108 | 100.00 | 0 | 0 | 0 | 0 |
|  |  | Baseline | 53 | 100.00 | 0 | 0 | 0 | 0 | 55 | 100.00 | 0 | 0 | 0 | 0 | 108 | 100.00 | 0 | 0 | 0 | 0 |
|  |  | Visit 1 | 53 | 100.00 | 0 | 0 | 0 | 0 | 54 | 98.18 | 1 | 1.82 | 0 | 0 | 107 | 99.07 | 1 | 0.93 | 0 | 0 |
|  |  | Visit 2 | 53 | 100.00 | 0 | 0 | 0 | 0 | 55 | 100.00 | 0 | 0 | 0 | 0 | 108 | 100.00 | 0 | 0 | 0 | 0 |
| Diastolic  blood pressure | | Screening | 52 | 98.11 | 1 | 1.89 | 0 | 0 | 53 | 96.36 | 2 | 3.64 | 0 | 0 | 105 | 97.22 | 3 | 2.78 | 0 | 0 |
|  |  | Baseline | 52 | 98.11 | 1 | 1.89 | 0 | 0 | 53 | 96.36 | 2 | 3.64 | 0 | 0 | 105 | 97.22 | 3 | 2.78 | 0 | 0 |
|  |  | Visit 1 | 51 | 96.23 | 2 | 3.77 | 0 | 0 | 55 | 100.00 | 0 | 0 | 0 | 0 | 106 | 98.15 | 2 | 1.85 | 0 | 0 |
|  |  | Visit 2 | 53 | 100.00 | 0 | 0 | 0 | 0 | 55 | 100.00 | 0 | 0 | 0 | 0 | 108 | 100.00 | 0 | 0 | 0 | 0 |
| Systolic blood pressure | | Screening | 50 | 94.34 | 3 | 5.66 | 0 | 0 | 49 | 89.09 | 6 | 10.91 | 0 | 0 | 99 | 91.67 | 9 | 8.33 | 0 | 0 |
|  |  | Baseline | 49 | 92.45 | 4 | 7.55 | 0 | 0 | 50 | 90.91 | 5 | 9.09 | 0 | 0 | 99 | 91.67 | 9 | 8.33 | 0 | 0 |
|  |  | Visit 1 | 50 | 94.34 | 3 | 5.66 | 0 | 0 | 52 | 94.55 | 3 | 5.45 | 0 | 0 | 102 | 94.44 | 6 | 5.56 | 0 | 0 |
|  |  | Visit 2 | 51 | 96.23 | 2 | 3.77 | 0 | 0 | 52 | 94.55 | 3 | 5.45 | 0 | 0 | 103 | 95.37 | 5 | 4.63 | 0 | 0 |
| Temperature | | Screening | 40 | 75.47 | 13 | 24.53 | 0 | 0 | 38 | 69.09 | 17 | 30.91 | 0 | 0 | 78 | 72.22 | 30 | 27.78 | 0 | 0 |
|  |  | Baseline | 39 | 73.58 | 14 | 26.42 | 0 | 0 | 40 | 72.73 | 15 | 27.27 | 0 | 0 | 79 | 73.15 | 29 | 26.85 | 0 | 0 |
|  |  | Visit 1 | 36 | 67.92 | 17 | 32.08 | 0 | 0 | 37 | 67.27 | 18 | 32.73 | 0 | 0 | 73 | 67.59 | 35 | 32.41 | 0 | 0 |
|  |  | Visit 2 | 34 | 64.15 | 19 | 35.85 | 0 | 0 | 31 | 56.36 | 24 | 43.64 | 0 | 0 | 65 | 60.19 | 43 | 39.81 | 0 | 0 |
| Respiratory rate | | Screening | 17 | 32.08 | 36 | 67.92 | 0 | 0 | 18 | 32.73 | 37 | 67.27 | 0 | 0 | 35 | 32.41 | 73 | 67.59 | 0 | 0 |
|  |  | Baseline | 21 | 39.62 | 32 | 60.38 | 0 | 0 | 19 | 34.55 | 36 | 65.45 | 0 | 0 | 40 | 37.04 | 68 | 62.96 | 0 | 0 |
|  |  | Visit 1 | 17 | 32.08 | 36 | 67.92 | 0 | 0 | 20 | 36.36 | 35 | 63.64 | 0 | 0 | 37 | 34.26 | 71 | 65.74 | 0 | 0 |
|  |  | Visit 2 | 25 | 47.17 | 28 | 52.83 | 0 | 0 | 22 | 40.00 | 33 | 60.00 | 0 | 0 | 47 | 43.52 | 61 | 56.48 | 0 | 0 |

*n*/%: number/percentage of patients with given characteristics.

**Table S8.** The study procedures/assessments performed at different visits.

| Tests and assessments | Screening  day 14 | Baseline  day 0 ± 5 | | Visit 1  Week 4  ± 5 days | Visit 2  Week 8  ± 5 days | Visit 3  Week 10  ± 5 days |
| --- | --- | --- | --- | --- | --- | --- |
| Screening | x | - | - | | - | - |
| Consent | x | - | - | | - | - |
| Enrolment | - | x | - | | - | - |
| Medical history | x | - | - | | - | - |
| Vitals^*^ | x | x | x | | x | - |
| Systemic examination | x | x | x | | x | - |
| Test request form (TRF) | x | - | x | | - | - |
| Patient diary | x | x | x | | x | - |
| Placebo (washout) | x | - | - | | - | - |
| Drug dispensing | - | x | x | | x | - |
| Drug returned | - | x | x | | x | - |
| Compliance | x^$^ | x | x | | x | - |
| Pain intensity assessment | - | x | x | | x | x |
| CSBM | - | x | x | | x | x |
| Severity of symptom domain score | - | x | x | | x | - |
| Stool consistency | - | x | x | | x | x |
| Physicians global assessment | - | x | x | | x | - |
| Patients global assessment | - | x | x | | x | - |
| Rescue medication record | - | x | x | | x | - |
| Concomitant medication record | - | x | x | | x | - |
| Adverse event monitoring | - | x | x | | x | x |
| Retrieval of unused drugs |  | x^#^ | x | | x | - |
| Review and retrieval of patient diary | - | x | x | | x | x |

x: Applicable at visit.

*: pulse rate, respiratory rate, blood pressure and temperature.

$: Run in period compliance review.

#: Retrieval of unused medication which also includes run in placebo.

**Table S9.** Prohibited medication.

| Agent | Mechanism of action | Targeted disorder |
| --- | --- | --- |
| Crofelemer | CFTR inhibitor | IBS-D |
| Linaclotide | Guanylate cyclase-c agonist | IBS-C |
| Arverapamil | Calcium channel blocker | IBS-D |
| Asimadoline | Kappa opioid agonist | IBS |
| Mitemcinal | Motilin receptor agonist | IBS-C |
| Ramosetron | 5-HT3 antagonist | IBS-D |
| TD-5108 | 5-HT4 agonist | IBS-C |
| DDP-773 | 5-HT3 agonist | IBS-C |
| DDP-225 | 5-HT3 antagonist/ NE reuptake inhibition | IBS-D |
| BMS-562086 | Corticotropin-releasing hormone antagonist | IBS-D |
| GW876008 | Corticotropin-releasing hormone antagonist | IBS |
| GTP-010 | Glucagon-like peptide | IBS pain |
| AGN-203818 | Alpha receptor agonist | IBS pain |
| Solabegron | Beta-3 receptor agonist | IBS |
| Espindolol (AGI-011) (324) | Beta receptor antagonist | IBS (all subtypes) |
| Dextofi sopam | 2,3 benzodiazepine receptors | IBS-D and IBS-M |

CFTR: cystic fibrosis transmembrane conductance regulator. IBS: Irritable bowel syndrome. IBS-C: constipation. IBS-D: diarrhea. IBS-M: Mixed. HT: hydroxytryptamine. NE: norepinephrine BMS: Bristol-Myers Squibb.

**Table S10.** Rescue medications.

| Symptoms | Generic name |
| --- | --- |
| Constipation | PEG 4000: (Miralax) 10 gm /day or  Lactulose: 1.3 g/kg/day both for two weeks and then  patients switched agents for a further two weeks1 |
| Diarrhea | Loperamide: ≤ 25 mg/kg/Day for diarrhea.  This should be done with oral rehydration solution (ORS)  unless the patient is vomiting or profoundly dehydrated |
| Antispasmodics | Peppermint oil: 1 ml three times a day |
